# Is in vitro micrografting a possible valid alternative to traditional micropropagation in Cactaceae? *Pelecyphora aselliformis* as a case study

**DOI:** 10.1186/s40064-016-1901-6

**Published:** 2016-02-27

**Authors:** Ornella Badalamenti, Angela Carra, Elisabetta Oddo, Francesco Carimi, Maurizio Sajeva

**Affiliations:** Institute of Biosciences and BioResources (IBBR), Research Division of Palermo, National Research Council (CNR), Corso Calatafimi 414, 90129 Palermo, Italy; Department of Biological, Chemical and Pharmaceutical Science and Technology (STEBICEF), University of Palermo, via Archirafi 18, 90123 Palermo, Italy

**Keywords:** CITES, Ex situ conservation, In vitro grafting, Micropropagation, Succulent plants, Tissue culture

## Abstract

Several taxa of Cactaceae are endangered by overcollection for commercial purposes, and most of the family is included in the Convention on International Trade in Endangered Species of Fauna and Flora (CITES). Micropropagation may play a key role to keep the pressure off wild populations and contribute to ex situ conservation of endangered taxa. One of the limits of micropropagation is the species-specific requirement of plant regulators for each taxon and sometimes even for different genotypes. With the micrografting technique the rootstock directly provides the scion with the necessary hormonal requirements. In this paper we present data on in vitro grafting of *Pelecyphora aselliformis* Ehrenberg, an Appendix I CITES listed species critically endangered and sought after by the horticultural trade, on micropropagated *Opuntia ficus*-*indica* Miller. Apical and sub-apical scions of *P. aselliformis* were used to perform micrografting with a successful rate of 97 and 81 % respectively. Survival rate after ex vivo transfer was 85 %. We hypothesize that this method could be applied to other endangered, slow growing taxa of Cactaceae thus contributing to the conservation of this endangered family.

## Background

Several taxa of Cactaceae are endangered by over-collecting for commercial purposes, and most of the family is included in the Convention on International Trade in Endangered Species of Fauna and Flora (CITES) (Sajeva et al. 2012). Usually the most endangered species are slow growing, not easily propagated and seeds may be rare and high priced. The request of the market is often fulfilled through illegal collection of wild specimens (Sajeva et al. 2013). Micropropagation may play a key role to keep the pressure off wild populations (Malda et al. 1999) and contribute to ex situ conservation. In vitro culture techniques allow massive propagation of several plant taxa starting from a small amount of plant material and with low impact on wild populations (Iriondo and Perez 1990) including Cactaceae among others (Lema-Rumińska and Kulus 2014). One of the limits of micropropagation is the species-specific requirement of plant growth regulators (PGR) for each taxon and sometimes even for different genotypes, besides the somaclonal variation which may be induced when a callus phase is present (Larkin and Scowcroft 1981).

Grafting is widely used to propagate several horticultural taxa (Hartmann et al. 1997), to overcome rooting problems and speed growth. With the grafting technique the scion growth requirements are provided directly from the rootstock. The use of in vitro grafting may enhance the advantages of both basic techniques, as shown in some woody species (e.g. Hassanen 2013; Niang et al. 2010).

In this paper we present data on in vitro micrografting of *Pelecyphora aselliformis* Ehrenberg, an Appendix I CITES listed species critically endangered and sought after by the horticultural trade, on micropropagated *Opuntia ficus*-*indica* Miller rootstock. We have tested if this method could be a more suitable alternative for propagation of this endangered species than traditional micropropagation as reported by Giusti et al. (2002).

## Methods

**Fig. 1:**
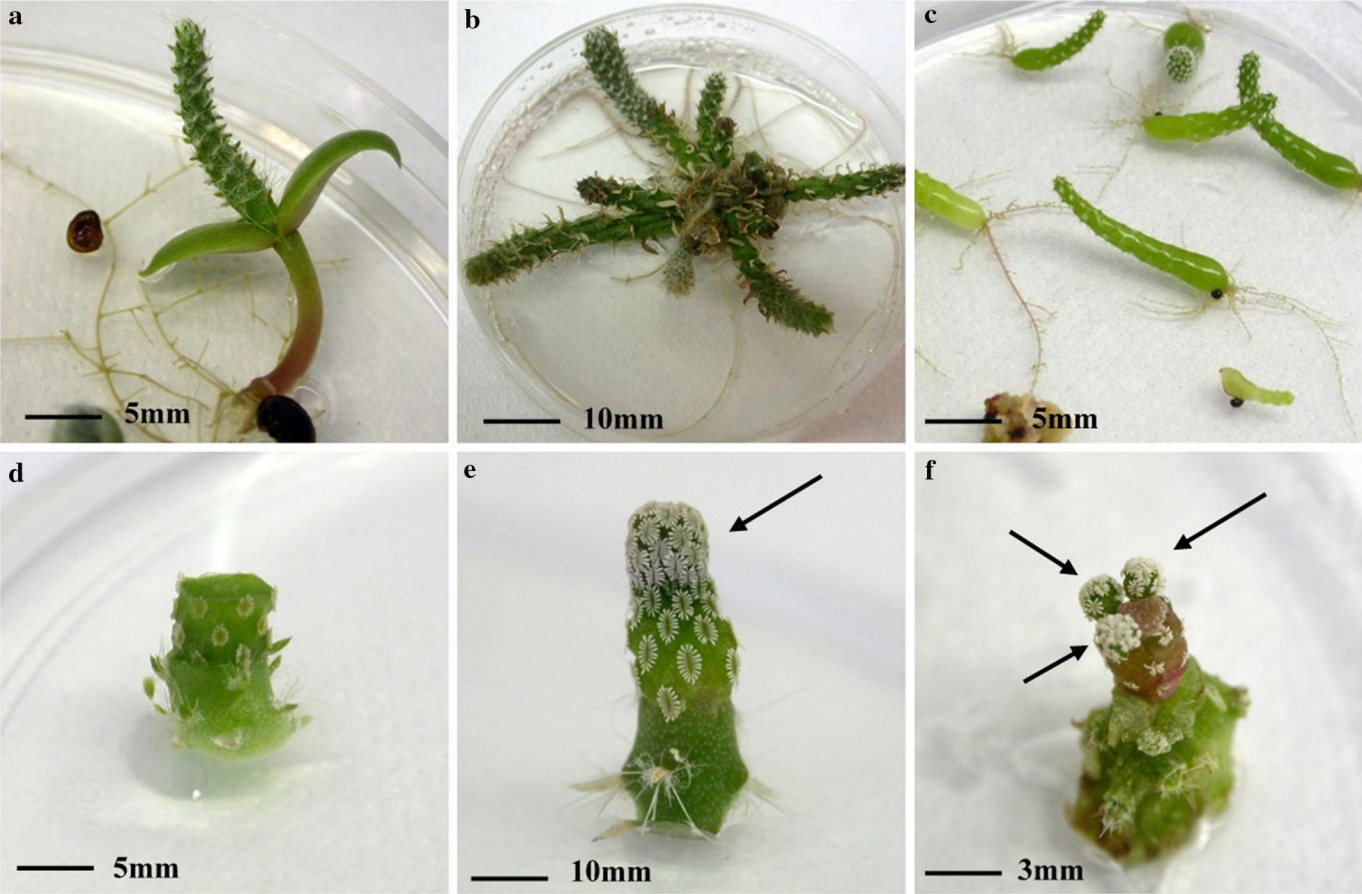
Different stages of micrografting procedures: **a** Seedling of *Opuntia ficus*-*indica* 30 days after sowing; **b** *Opuntia ficus*-*indica* subcultured and maintained as rootstock source indefinitely; **c** Seedlings of *Pelecyphora aselliformis* 60 days after sowing; **d** Freshly grafted sub-apical slice of *Pelecyphora aselliformis* on micropropagated *Opuntia ficus*-*indica*; **e** Micrografting of the apical part of *Pelecyphora aselliformis* after 20 days of cultivation. *Arrow* indicates new growth; **f** Micrografting of a sub-apical slice of *Pelecyphora aselliformis* after 20 days of cultivation. *Arrows* indicate new shoots arising from three different areoles

Seeds of *O. ficus*-*indica* were obtained from a plant cultivated at the Botanic Garden of Palermo, Italy. Seeds of *P.aselliformis* were purchased from Koehres Kakteen (Germany). To establish the in vitro cultures, sixty seeds of *O. ficus*-*indica* were chemically scarified with concentrated H_2_SO_4_ for 15 min and rinsed three times under aseptic conditions with sterile distilled water (SDW) and germinated on MS medium (Murashige and Skoog 1962) supplemented with 146 mM sucrose and 1 µM gibberellic acid (GA_3_) (pH 5.7 ± 0.1). Seedlings of *O. ficus*-*indica* (Fig. 1a) were used as starting material to obtain suitable micropropagated rootstock for in vitro micrografting. Preliminary experiments showed that the best results in terms of rootstock production were obtained when explants were cultured in presence of 2 µM 6-benzylaminopurine (BA). This plant material was subcultured every 30 days and maintained as rootstock source indefinitely (Fig. 1b).

Forty-five seeds of *P. aselliformis* were disinfected for 1 min in 70 % ethanol, 25 min in 2 % sodium hypochlorite (w/v), rinsed four times under aseptic conditions with SDW and germinated on MS medium (Fig. 1c).

All cultures were maintained in a climate chamber at 25 ± 1 °C under a 16 h day length, and a photosynthetic photon flux of 50 µmol m^−2^ s^−1^ provided by Osram cool-white 18 W fluorescent lamps.

Micrografts were carried out using *O. ficus*-*indica* as rootstock and *P. aselliformis* seedlings as scion. Micrografting procedures were based upon those described by Estrada-Luna et al. (2002) with few modifications. Micrografts were performed on 10 mm long fresh cut rootstock of *O. ficus*-*indica* without roots. Shoots were cut under aseptic conditions at the base and at the top with a single stroke of a razor blade. The scion was cut into apical and sub-apical segments (slices) of 5 and 3 mm, respectively. To maximize the production of slices no areoles were left in the remaining hypocotyl. The remaining part was discarded.

The freshly cut scion was brought in contact with freshly cut rootstock tissue (Fig. 1d), the two bionts were incubated on medium to induce root formation. Periodically, axillary shoots that emerged from rootstocks were removed. The different steps of micrografting were carried out on PGR free MS medium and maintained as described above. Micrografts were done in separate batches. When sub apical explants were used, each set comprised 90 micrografts while, when apical explants were used, each set was of ten micrografts.

Well developed micrografts 25 mm long were collected and washed in SDW to remove the medium, then transferred into autoclaved Jiffy 7^®^ peat pellets soaked in 40 ml of SDW. After transplanting, plants were maintained under 95 ± 5 % relative humidity in a growth chamber till roots grew out of the pellet, and then transferred to the greenhouse, exposed to daylight conditions at 25 ± 2 °C day, 20 ± 2 °C night. Micrografting response was quantified by the number of non-necrotic scions and the number of offsets produced by the scions 60 days after micrografting. Scion growth was evaluated by recording the mean number of new shoots and the shoot length 3 and 6 weeks after grafting.

## Results

Germination of *O. ficus*-*indica* started about 30 days after sowing (Fig. 1a) and germination frequency was 75 % after three months. Successfully germinated seedlings (45) reached 30–40 mm in length within 3 months after germination with a mean diameter of the epicotyl of 5 mm. Plantlets of *O. ficus*-*indica* produced the first shoots from the areoles 15 days after culture initiation, and after 30 days the mean number of shoots produced from areoles was 7.4 per explant with a mean shoot length of 17 mm (Fig. 1b).

Germination of seeds of *P. aselliformis* occurred gradually starting 20 days after sowing and germination frequency was 73.3 %. Successfully germinated seedlings (33) reached 40 mm in length within 3 months after germination with a mean diameter of 5 mm. Seedlings were considered suitable for micrografting when they were at least 10 mm long (Fig. 1c).

To maximize the productivity, sub-apical slices of *P. aselliformis* 3 mm in length were used for grafting (Fig. 1d). Each slice had at least one areole, which potentially can produce new shoots. Preliminary experiments showed that rootstock with a diameter between 4 and 6 mm gave the best results in grafting compatibility. In total we used 30 apical scions (≈150 mm total length) and 270 sub-apical slices corresponding to ≈810 mm, excluding the part below the cotyledons which has no areoles.

No problems were observed during union formation and only a small percentage (10 %) of graft failure occurred due to scion displacement. 81 % of micrografts were successful and gave rise to new individuals. The re-establishment of growth of the scion was evident 3 weeks after micrografting (Table 1; Fig. 1e–f). Six weeks after micrografting 90 % of successful micrografts developed roots. When the mean length of the scion ranged from 35 to 40 mm grafted plants were considered suitable for a new multiplication cycle or for in vivo transfer. For in vivo transfer, grafts with a well developed root apparatus were transplanted in Jiffy pots and 2 months after the in vivo transfer, the survival rate was of 85 % (Table 1).

**Table 1:** Success rate and growth of micrografted plants of *P. aselliformis* on micropropagated *O. ficus indica* rootstock. Mean ± SE

Type of scion	No. of micrografts	Successful micrografts (%)	Scion growth			Rooted micrograft (%)	Successful ex vivo transfer (%)
			Mean No. of shoots	Shoot length (mm)^a^	Shoot length (mm)^b^		
Sub-apical	270	81 ± 1.2	2.7 ± 0.3	28 ± 0.4	35 ± 0.5	90 ± 1.2	85 ± 0.8
Apical	30	97 ± 0.7	1.0 ± 0	31 ± 0.6	38 ± 0.3		

^**a**^Three weeks after micrografting

^**b**^Six weeks after micrografting

## Discussion

The use of PGR may induce somaclonal variation in different regeneration systems in vitro, as reported for many species, including several members of Cactaceae (Corneanu et al. 1990; Mangolin et al. 1994). Our results show that by micrografting it is possible to propagate *P. aselliformis* in vitro without adding combinations of PGR to the culture medium. In this way there is no need to test the genetic stability of regenerants. Additionally, it is not necessary to carry out time-consuming preliminary tests to find the ideal PGR requirements for micropropagation, or to analyse the endogenous hormonal content as in Sriskandarajah et al. (2006) because the rootstock directly provides the scion with the required hormones (Webster 1994). Moreover the use of PGR often induces callus production from which shoots emerge with a very high incidence of hyperhydricity (Giusti et al. 2002). This is a further reason for which the establishment of a multiplication procedure with no PGR added should be preferred for endangered plants.

Our in vitro cultured plants showed a faster growth rate compared to soil-grown ones, as reported by Giusti et al. (2002). The number of grafted plants produced was two per centimetre of seedling for the first propagation cycle, which was less than that obtained by Giusti et al. (2002); however, it was possible to produce plants with no hyperhydricity and avoid the need of preliminary in vitro proliferation cycles as described by Pérez-Molphe-Balch and Dàvila-Figueroa (2002). Furthermore, the newly obtained plants could be used for subsequent propagation cycles.

These results clearly show that it is possible to micrograft small apical and sub-apical slices (5 and 3 mm, respectively) of *P. aselliformis* onto *O. ficus*-*indica* rootstock, obtaining a good percentage of successful grafts and the production of new specimens. Furthermore in vitro grafted plants may constitute a suitable tool for ex situ conservation. On-going research on different species of Cactaceae is testing if this method is suitable for other endangered species. The micrografting technique fulfils the definition of artificial propagation as requested by CITES and does not cause damage to the wild populations of the species and therefore can be considered “non detrimental” for the wild populations.

## Conclusions

This study describes the protocol for micrografting of an endangered Cactaceae, *Pelecyphora aselliformis.* Our results indicate that it is possible to start massive propagation with just a few seeds without the need to find the optimal in vitro requirements, which are species-specific, and to some extent genotype-specific. This technique could further be useful for the massive production of ornamental plants reducing the overharvesting of endangered species from the wild. Moreover, the production of micrografted plants may contribute also to ex situ conservation of this threatened species.
